# Women with perinatal suicidal ideation–A scoping review of the biopsychosocial risk factors to inform health service provision and research

**DOI:** 10.1371/journal.pone.0274862

**Published:** 2022-09-28

**Authors:** Ann-Marie Bright, Owen Doody, Teresa Tuohy

**Affiliations:** Department of Nursing and Midwifery, University of Limerick, Limerick, Ireland; Nazarbayev University School of Medicine, KAZAKHSTAN

## Abstract

**Objectives:**

This review aims to map the existing evidence on perinatal suicidal ideation, identify biopsychosocial risk factors associated with suicidal ideation and make recommendations for service provision and future research.

**Methods:**

Scoping review guided by Arskey’s and O’Malley’s (2005) framework. Five academic databases (PsycINFO, MEDLINE, CINAHL, ASSIA and Academic Search Complete) were searched from 1^st^ January 2009 to 1^st^ April 2022. Studies were screened by title, abstract and full text against inclusion and exclusion criteria. Primary qualitative, quantitative and mixed-methods studies, written in English pertaining to perinatal suicidal ideation were included. Forty-one studies met the eligibility criteria, data were extracted and narratively synthesised. Findings are reported in accordance with the PRISMA-SR extension.

**Key conclusions:**

Findings were mapped onto the biopsychosocial framework and include sleep deprivation, maternal age, pregnancy complications, mood disorders, intimate partner violence, childhood maltreatment/abuse, low socioeconomic status, alcohol and tobacco misuse, miscarriage/perinatal loss, birth trauma and sleep deprivation. The findings demonstrate that the biopsychosocial risk factors for perinatal suicidal ideation are varied and complex.

**Implications for practice:**

The minimisation of women’s experiences may lead to detrimental consequences and there is a need for increased knowledge of mental health problems by those working with women in the perinatal period to ensure safety planning conversations occur with every woman meeting ‘at risk’ criteria.

## Introduction

The perinatal period is defined as the time encompassing pregnancy through to the first year after birth when women are vulnerable to distress and the risk of developing or having a recurrence of mental health problems [1, 2]. Antenatal anxiety and depression are reported to occur in up to 33% of pregnancies [3]. The antenatal period is also a time when up to 40% of postnatal depressive episodes begin [4], a condition with a global prevalence range of 0.5% to 60% [5]. The biopsychosocial impacts of pregnancy and birth as well as the transition to motherhood must be considered when treating perinatal depression [6, 7] as there is a growing body of knowledge highlighting the positive association between maternal perinatal mental health problems and psychological disturbances in children and families [8–10].

Suicidal ideation is common among individuals experiencing mental health problems, defined as preoccupation with death by suicide; it differs significantly to self-harm or non-suicidal self-injury, where the individual’s intent is to reduce or signal to others, they are experiencing stress but not to die [11–13]. In essence, suicidal ideation is a prodromal state, where the individual is contemplating or planning their death by suicide. Therefore, it is imperative to identify those at risk of suicidal ideation to offer support and interventions before suicide is completed. The International Classification of Diseases-10 (ICD-10) [14] categorises suicide, in the context of maternal morbidity, as a direct cause of death [15]. The United Kingdom (UK) and Ireland’s Confidential Enquiries into Maternal Deaths and Morbidity 2014–2016 and 2016–2018 found suicide to be the leading direct cause of death for up to one year after birth [15, 16], a finding mirrored in other countries such as France [17] and Australia [18].

Staff knowledge and understanding of perinatal mental health, specifically those working in primary care or maternity services, is essential for identifying women at-risk of suicidal ideation [1]. Reports suggest healthcare practitioners working in primary care enquire about mood disorders, substance/alcohol use, anxiety and past experiences but are less likely to ask about suicidal ideation [1]. Therefore, those experiencing what are considered ‘psychiatric emergencies’ are at risk of not being recognised or identified [1]. To date, reviews have focused on the prevalence rates of self-harm [19] and suicidal ideation [20] among perinatal populations and the role of the midwife in suicide prevention [21]. A mini review was conducted by Orsolini *et al*. [22] however its focus was suicidal epidemiology, risk factors and clinical correlates and not specifically risk factors for suicidal ideation. O’Connor *et al*. [23] conducted a systematic review examining the risk factors associated with suicidal ideation in the perinatal period and what screening tools were used to identify perinatal suicidal ideation. Nonetheless, the data search within O’Connor *et al*.*’s* [23] review was limited to quantitative studies published in a five-year period spanning 2013–2018 and whilst the review provides a comprehensive assessment of the eligible studies, it captures only a narrow focus of the existing literature. Reid *et al*. [24] conducted a systematic review on maternal suicide ideation in the perinatal period to identify psychological and psychosocial risk factors. However, Reid *et al*. [24] excluded studies conducted on mothers who were aged <18 and studies conducted on mothers who experienced pregnancy loss and again, whilst a comprehensive assessment of the eligible studies was presented, a consequence of the exclusion criteria applied means pertinent and relevant data may have been omitted. Therefore, to identify the range and extent of studies pertaining to perinatal suicidal ideation [25] and to make recommendations for practice and research [26] this scoping review focuses on “*what are the reported biopsychosocial risk factors associated with suicidal ideation during the perinatal period*?*”*

## Methods

Arksey and O’Malley’s [27] framework for scoping studies informed the procedures for this review involving a five-step process; i.) identifying the research question; ii.) identifying relevant studies; iii.) study selection; iv.) charting the data and v.) collating, summarising and reporting the results. Scoping reviews allow for general exploration of diverse studies and whilst not considered as high-level as systematic reviews, are considered to exist at a higher level than that of integrative or literature reviews [28]. To improve the quality of reporting in scoping reviews, the PAGER framework [26] was utilised to i.) identify patterns in the data; ii.) identify gaps in the data; iii.) highlight evidence for practice from the data; and iv.) give recommendations for research. To guide the review, inclusion and exclusion criteria were identified. Studies were included if they i.) elicited the perspectives or narratives of women experiencing suicidal ideation in the perinatal period or ii.) they elicited quantitative data relevant to suicidal ideation during the perinatal period. Studies were excluded if they i.) focused on completed suicide or ii.) participants were engaged in self-harm behaviours. The search was limited to peer-reviewed articles published between 1^st^ January 2009 and 1^st^ April 2022; 2009 was chosen as this was the date of inception for the Maternal Death Enquiry Ireland report [29]. This review considered all studies that had used qualitative, quantitative and mixed-method’s designs.

A Population, Interest, Context (PIC) framework was used to inform the choice of keywords in the search strategy. A preliminary search of Cumulative Index to Nursing and Allied Health Literature (CINAHL) and Academic Search Complete (ASC) was conducted to trial keywords and index terms. During these initial scoping searches, it was observed that using a detailed facet analysis resulted in the return of irrelevant studies. Therefore, subsequent scoping searches were used with more refined terms and resulted in the return of more relevant studies. Five academic databases PsycINFO, Applied Social Sciences Index and Abstracts (ASSIA), MEDLINE, CINAHL and Academic Search Complete were searched on 1^st^ April 2022 using the identified keywords and search terms outlined in Table 1. Backward chaining involving hand searching of the reference lists from returned studies was conducted to identify additional studies that may have been overlooked during the search. Grey literature was omitted to ensure a transparent and replicable search of peer reviewed literature [30]. Whilst it is acknowledged the omission of grey literature may introduce a publication bias, the difficulty in determining whether grey literature is peer-reviewed offsets this risk.

**Table 1:** PIC framework and search terms.

**PIC Framework**	**Search terms**	**Search**
Population	Mother* OR Matern*	Search 1
Interest	Perinatal OR antenatal OR ante-natal OR postnatal OR post-natal	Search 2
Context	Suicid* OR “suicidal ideation”	Search 3
		S1 + S2 + S3 ‘search with AND’ = results

**Fig 1:**
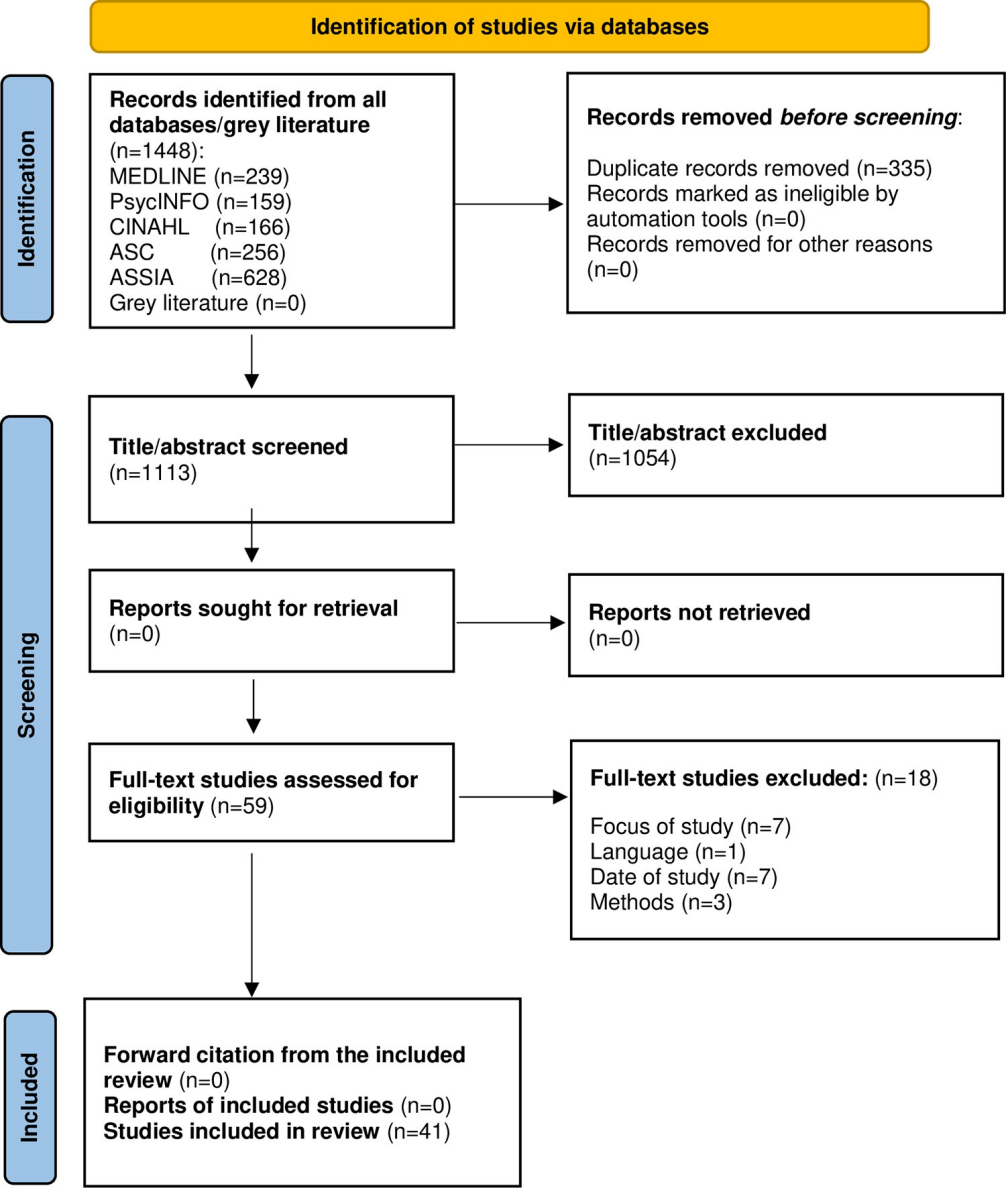
Prisma flow diagram (Page et al. 2021).

The search generated 1448 returns and duplicates (n = 335) were removed. Remaining citations (n = 1113) were screened by title and abstract resulting in (n = 59) going forward for full-text screening. A total of forty-one studies (n = 41) were included for review, an overview is provided in Fig 1 [31]. The primary author (AMB) extracted the findings and discussed findings with the other authors (TT, OD). Data were extracted in accordance with the research question using a data extraction table that was agreed by all authors and addressed the following: author, year, country of origin, sample size, study aim/hypothesis, methods, data analysis, participant characteristics, findings relevant to suicidal ideation in the perinatal period, data management, reported themes and subthemes and conclusions and recommendations. Any disagreements were resolved through discussion and consensus agreement between all authors. Studies were read and re-read, and findings charted [27]. Narrative synthesis was conducted guided by Popay *et al*. [32]. In line with the purpose of scoping reviews this review identifies, maps and charts data and quality appraisal or risk of bias assessments were not completed as the focus is on description.

## Findings

The findings from this review are reported in accordance with the Preferred Reporting of Items for Systematic reviews and Meta-Analyses (PRISMA) Scoping Review extension checklist (see S1 Checklist) [25]. Through the data extraction and synthesis process, all data were mapped onto the objectives of this review and findings were grouped in accordance with the biopsychosocial framework, that suggests biological, psychological and social factors and their interactions should be considered when attempting to understand an individual’s presentation [33].

### Characteristics of studies

Of the forty-one studies that met eligibility criteria, twelve were conducted in the USA [34–45], five in Ethiopia [46–50], four in South Africa [51–54], three in Italy [55–57], two in China [58, 59], two in Japan [60, 61], one in Peru [62], one in the UK [63], one in France [64], one in Canada [65], one in Germany [66], one in Spain [67], one in Romania [68], one in Bangladesh [69], one in Pakistan [70], one in India [71], one on the Thailand-Myanmar border [72], one in Tanzania [73] and one in Ireland [74]. In total, 55,439 participants were recruited across the forty-one studies with individual sample sizes ranging from 14 [37] to 22,118 [36]. An overview of the study characteristics is provided in Table 2.

**Table 2:** Study characteristics.

Author, Year & Country	Title of study	Aim/Objective	Design/Methodology	Number of participants in study	Outcome measures used within the study	Key findings
Achtyes *et al*. (2020) USA	Inflammation and kynurenine pathway dysregulation in post- partum women with severe and suicidal depression.	To assess the immunobiological pathways in women with severe and suicidal depression.	Cross-sectional study. Social Sciences (v 23 for Mac).	168	Edinburgh Postnatal Depression Scale (EPDS).	Depressive symptoms and suicidal behaviour were linked to inflammation and low levels of serotonin in peripheral blood samples.
Alhusen *et al*. (2015) USA	Intimate partner violence and suicidal ideation in pregnant women.	To examine the prevalence of SI and comorbid depressive symptomatology during pregnancy. Identify risk factors for SI during pregnancy in low-income predominantly African-American sample of pregnant women.	Cross-sectional study. Chi-square. Student t-test. STATA 11.	166	Edinburgh Postnatal Depression Scale (EPDS), Abuse Assessment Screen.	Nearly 20% experienced abuse in their current pregnancy. N = 38 endorsed SI in current pregnancy. Of those experiencing SI, 97.3% had EPDS >12. Women who endorsed SI were more likely to have less than 12 years formal education, less than $10,000 annual income, living without a partner or spouse and experiencing IPV. Clinically significant depressive symptoms remained significant factor for SI. IPV also significant predictor of SI–associated with over 9x increased odds of SI. IPV independent risk during pregnancy for SI after controlling for depressive symptoms.
Ammerman *et al*. (2019) USA	Interpersonal trauma and suicide attempts in low-income depressed mothers in home visiting.	To describe the clinical and psychosocial features of low-income mothers with major depressive disorder (MDD) who were enrolled in home visiting and who had made a suicide attempt at some point in their lives.	No study design reported. MANOVA Cohen’s d effect Mplus v 6.12	170	Suicide History Questionnaire (SHQ), Structured Clinical Interview for DSM-IV Axis 1 Disorders (SCID), Beck Depression Inventory-ii (BDI-II), Childhood Trauma Questionnaire (CTQ), Composite Abuse Scale (CAS), Posttraumatic Stress Disorder Checklist (PCL-S), Interpersonal Support Evaluation (ISEL), Social Network Index (SNI).	Suicide attempt mothers had more MDD symptoms, episodes and younger age at the time of the MDD episode. Suicide attempt group more likely to have recurrent MDD as opposed to single episode. Suicide attempts group reported higher levels of childhood trauma history Suicide attempt mothers more likely to meet criteria for PTSD Suicide attempt mothers more likely to have less social support and smaller social networks.
Anbesaw *et al*. (2021) Ethiopia	Suicidal ideation and associated factors among pregnant women attending antenatal care.	To explore the prevalence of suicidal ideation and associated factors among pregnant women attending antenatal care in Jimma, Ethiopia.	Cross-sectional study. Epi-Data v 3.1 SPSS 25.0	415	Edinburgh Postnatal Depression Scale Depression (EPDS), Anxiety and Stress Scale (DASS), Abuse Assessment Scale (AAS), Childhood Physical and Sexual Abuse questionnaire, Perceived Stress Scale (PSS), Maternity Social Support Scale (MSSS), Pittsburgh Sleep Quality Index (PSQI), World Mental Health survey (WMH), Student drug use questionnaire.	Marital status income, parity, educational status, history of abortion, unplanned pregnancy, family history of mental illness, family history of suicide attempt, depression, anxiety, poor sleep quality, stress, intimate partner violence and social support identified in bivariate analyses. Marital status, depression, anxiety, history of absorption, sleep quality, stress and intimate partner violence statistically significant in multivariate analyses. Single, widowed, divorced = 2.8 times more likely to experience SI. History of abortion = 2.45 times more likely to experience SI. Depression = 4times more likely to experience SI. Anxiety = 3 times more likely to experience SI. Stress = 2.50 times more likely to experience SI. IPV 2.43 times more likely to experience SI.
Asad *et al*. (2010) Pakistan	Prevalence of suicidal thoughts and attempts among pregnant Pakistani women.	To determine the prevalence of suicidal thoughts and attempts and to identify demographic variables and mental health correlates such as anxiety/ depression and domestic violence among pregnant women in an urban community in Pakistan.	Cross-sectional study. SAS v 9.2.	1369	Aga Khan University Anxiety and Depression Scale (AKUADS).	Education, depressive symptoms were associated with suicidal thoughts. Formal/informal education, depression/anxiety, verbal abuse and physical/sexual abuse were associated with suicide attempts. Strong associations between suicidal thoughts for those who experienced abuse once a week.
Belete *et al*. (2021) Ethiopia	Prevalence and correlates of suicide ideation and attempt among pregnant women attending antenatal care services at public hospitals in Southern Ethiopia.	To assess the prevalence and factors associated with suicide ideation and attempt among pregnant women attending antenatal care services at public hospitals in southern Ethiopia.	Cross-sectional study. SPSS v 20. Chi-square test.	762		Age, marital status, monthly income, gestational age, gravidity, unwanted pregnancy, unplanned pregnancy, lifetime SI, social support, alcohol use, common mental health disorders, past history of physical violence and current physical violence were identified in bivariable analyses. Unplanned pregnancy, lifetime SI, poor social support and common antenatal mental disorders significantly associated with SI during current pregnancy in multivariate analyses.
Belete and Misgan (2019) Ethiopia	Suicidal behaviour in postnatal mothers in northwestern Ethiopia: a cross-sectional study.	To assess the prevalence and associated factors of suicidal behaviour (suicidal ideation, plan or suicide attempt) in postpartum mothers.	Cross-sectional study. EpiData v 3.1. SPSS v 2.0.	988	Suicidal Screening Tool, Mini-international Neuropsychiatric Interview, Oslo-3 Social Support Scale, Alcohol, Smoking and Substance Involvement Screening Test, Abuse Assessment Scale.	Educational status, sexually unfaithful husband, poor wealth of the mother, unplanned pregnancy of current child, verbal abuse, history of rape and sickness of infant identified in bivariate analyses. Poor wealth of mother, unplanned pregnancy of current child, history of rape and sickness of infant statistically significant in multivariate analyses.
Bodnar-Deren *et al*. (2016) USA	Suicidal ideation during the postpartum period.	To investigate the prevalence of SI in the 1^st^ 6 months postpartum among a sample of relatively healthy women and to identify baseline sociodemographic, clinical and psychosocial characteristics associated with later SI. Examine whether self-report symptoms of depression and/or anxiety assessed 24–48 hours after delivery were associated with later SI.	Retrospective study. Chi-square. Fishers exact test. Students t-test. SAS v 9.2.	1073	Edinburgh Postnatal Depression Scale (EPDS), Patient Health Questionnaire (PHQ), Generalised Anxiety Disorder 7 scale (GAD-7).	Later SI was significantly associated with race/ethnicity, being Spanish-speaking, foreign born and antepartum complications. Bivariate analyses revealed baseline depression and anxiety screenings were significantly associated with later SI. History of depression was correlated with SI.
Du toit *et al*. (2020) South Africa	Perinatal suicidality: Risk factors in South African women with mental illness.	To assess suicidality and associated factors during pregnancy and the postpartum period amongst women with known psychiatric diagnoses.	Quantitative-descriptive study. No statistical package reported.	263	Mini International Neuropsychiatric Interview (INI), Montgomery Asberg Depression Rating Scale (MADRS).	Significantly higher risk of experiencing suicidality if there was a suicide attempt before pregnancy. Late presentation (>27 weeks) to mental health clinics was a highly significant risk for suicidality. Women who were unemployed and had unwanted pregnancies had a significantly higher risk of suicidality. Presence of MDD and BPT positively associated with suicidality whilst schizophrenia spectrum disorders and GAD negatively associated with suicidality.
Doherty *et al*. (2019) Ireland	Suicidality in women with adjustment disorders and depressive episodes attending an Irish Perinatal Mental Health Service.	To examine the presenting symptoms of women attending perinatal psychiatry services at a Dublin maternity hospital who are diagnosed with adjustment disorder or depressive episodes and compare them to a comparison group recruited from liaison psychiatry.	Post-hoc analysis. Students t-test. Chi-square. SPSS v.20.	45	Schedules for clinical assessment in neuropsychiatry, IDS-C30 –Inventory of Depressive Symptoms Clinician Rated, The list of threatening experiences, standardised assessment of personality abbreviated scale, OSLO social support scale, Suicide intent scale. Scale of suicidal ideation.	22% reported SI or behaviours which were more common in women with a diagnosis of AD or DE although not statistically significant. 3 participants with suicidal behaviours– 2 had AD; 1 DE. AD had higher levels of intent although not statistically significant. No differences observed in social support/social functioning. Participants with AD had x2 the mean numbers of stressful life events compared to those with DE. No statistically significant differences in depressive symptoms between both groups.
Enatescu *et al*. (2020) Romania	The role of personality dimensions and trait anxiety in increasing the likelihood of suicide ideation in women during the perinatal period.	To identify the presence of suicide ideation among pregnant women who gave birth in our region. The secondary objectives were to explore the personality traits and demographic characteristics that are significantly correlated with and increased likelihood of developing suicide ideation in perinatal women.	Longitudinal prospective study. SPSS v 17. Chi-square tests. Kendall’s Tau-b correlation coefficient.	213	Edinburgh Postnatal Depression Scale (EPDS), State and Trait Anxiety Inventory (STAI-Y), NEO-FFI.	Lower education and employment was highlighted in mothers presenting with SI. Of all women presenting with SI only (n = 24) of antenatally assessed and (n = 6) of postnatally assessed mothers also had significant scores for depression. (n = 12) of pregnant women and (n = 6) of postnatal women also presented with state anxiety symptoms. Neuroticism higher in postnatal women with SI. Conscientiousness had a predictive value for antenatal SI. Postnatal trait anxiety was predictive on postnatal SI.
Fellmeth *et al*. (2021) Thailand- Myanmar	Suicidal ideation in the perinatal period: findings from the Thailand- Myanmar border	To report data on suicidal ideation from a cohort of migrant and refugee women living in the area between 2015 and 2017.	Prospective cohort study. No statistical package reported.	568	Structured Clinical Interview–DSM-IV (SCID).	Suicidal ideation was higher among refugee women (8%) than migrant women (3%).
Garman *et al*. (2019) South Africa	Association between perinatal depressive symptoms and suicidal risk among low-income South African women: a longitudinal study.	To assess the association between depressive symptoms and suicidal risk over time among perinatal women at risk for depression antenatally, and asses modifying effects of age, perinatal stage and depressive symptom trajectory.	Longitudinal study. STATA v 14.	425	Mini-international Neuropsychiatric Interview (MINI), Hamilton Depression Rating Scale (HDRS).	Change in HDRS scores was associated with suicide outcomes after controlling for age and trajectory. One point change in HDRS score was associated with a 0.68point change in suicide score among younger and middle-age participants but not the older group. Suicide risk was 1.17 times greater with every one-point increase in HDRS score among younger and middle-ages participants but not older participants.
Gelabert *et al*. (2020) Spain	The role of personality dimensions, depressive symptoms and other psychosocial variables in predicting postpartum suicidal ideation: a cohort study.	To examine the prevalence of postpartum SI in a large cohort of healthy Spanish women from the general population within 32 weeks of childbirth. To study Eyseneck’s Personality Dimensions (neuroticism, psychoticism, extraversion) as risk factors for postpartum SI, considering depression and other variables (psychiatric history, social support and stressful life events).	Cohort study. Chi-square. Fishers exact test. Student’s t-test. SPSS V.19.0.	1337	Edinburgh Postnatal Depression Scale (EPDS), Diagnostic Interview for Genetics Studies, Eyesnick’s Personality Questionnaire–revised short scale, Duke-UNC–functional social support questionnaire, St. Paul Ramsey Life Experience Scale, Semi-structured interview.	Women with SI in early postpartum, 8 weeks and 32 weeks showed higher scores on neuroticism and psychoticism. High prevalence of postpartum SI and the strong association between SI and depression in the postpartum. Risk factors = high neuroticism, psychoticism, psychiatric history, early depressive symptoms and having experienced at least one stressful life event during pregnancy.
Girardi *et al*. (2011) Italy	Temperament, post-partum depression, hopelessness and suicide risk among women soon after delivering	To assess the prevalence of PPD in a sample of young women and to evaluate the association between affective temperaments and emotional disorders	Cross-sectional study. Two-step cluster analyses. One-way fisher exact test. Chi-square. Student t-test. SPSS 13.0. ANOVAS.	92	Edinburgh Postnatal Depression Scale (EPDS), Beck Hopelessness Scale, Gotland Male Depression Scale, Suicidal History Self-Rating Scale.	Cluster 1 –low temperament traits = depression, cyclothymia, irritability and anxiety. Cluster 2 –higher scores on depression, cyclothymia, irritability and anxiety traits–associated with dysphoric/dysregulated temperaments. Women in cluster 2 had higher mean scores on BHS, GMDS, EPDS & SHSS. 52% of those in cluster 2 had EPDS >13, compared to 8% in cluster 1. None attempted suicide but previous findings have demonstrated personality and affective temperament traits may play a role in suicide.
Gressier *et al*. (2016) France	Risk factors for suicide attempt in pregnancy and the post-partum period in women with serious mental illnesses	To identify independent risk factors for suicide attempt in pregnancy and/or the postpartum among women with a psychiatric disorder hospitalised in the year following childbirth in a mother-baby unit.	Retrospective study. R v. 2.14.1 Kruskal Wallis test. Chi-square. Gibb sampler.	1439	Maternal sociodemographic characteristics, Marce Clinical Checklist.	Women with SA in pregnancy and postpartum were significantly younger. Miscarriage, alcohol use, smoking during pregnancy, poor family/social support were significantly more frequent when SA occurred in pregnancy. A diagnosis of MDD was significantly more frequent with SA occurred in postpartum. History of miscarriage and tobacco use during pregnancy were independently associated with SA in pregnancy. Younger maternal age, psychiatric diagnosis of MDE or recurrent depression independently associated with SA in postpartum. Alcohol and tobacco use in pregnancy is associated with increased risk of SA during pregnancy.
Howard *et al*. (2011) UK	The prevalence of suicidal ideation identified by the Edinburgh Postnatal Depression Scale in postpartum women in primary care: findings from the RESPOND trial.	To determine the prevalence of SI as measured by the EPDS in a primary care population of women at 6–8 weeks postpartum, validate EPDS measure of SI using clinical interview schedule, determine risk factors associated with EPDS measure of suicidality, examine whether SI is associated with worse outcomes.	Prospective cohort study forming part of RCT. Students t-test. Chi-square test. No statistical package reported.	253	Edinburgh Postnatal Depression Scale (EPDS).	Women more likely to experience SI at baseline if they were younger, unmarried, unemployed or had a partner that was unemployed. Multivariate analysis–significant for age, EPDS & >3 children.
Islam *et al*. (2020) Bangladesh	Do maternal depression and self-esteem moderate and mediate the association between intimate partner violence after childbirth and postpartum suicidal ideation?	To examine the association of experiencing physical, psychological and sexual IPV after childbirth with postpartum SI. To examine whether postpartum depressive symptoms and self-esteem act to mediate or moderate the relationship between IPV and postpartum SI.	Cross-sectional study. SPSS v 22.0.	426	Edinburgh Postnatal Depression Scale (EPDS), Rosenberg Self-Esteem Scale (RSES), Family Needs Screener	Women experiencing suicidal ideation were more likely to have reported psychological, physical and sexual IPV after childbirth. Physical, sexual and psychological IPV all significantly linked with postpartum SI.
Kalmbach *et al*. (2020) USA	Depression and suicidal ideation in pregnancy: exploring relationships with insomnia, short sleep and nocturnal rumination.	To explore the associations among sleep symptoms, rumination, depressive symptoms and suicidal ideation. Predicted that pregnant women with insomnia and high nocturnal rumination would be at highest risk of endorsing major depression and SI. Predicted women with insomnia and short sleep would be at highest risk of endorsing depression/suicidality than other groups.	Part of a larger Randomised Control Trial. SPSS v.25 Student’s t-test.	267	Insomnia Severity Index, Pittsburgh Sleep quality Index, Edinburgh Postnatal Depression Scale, Pre-sleep Arousal Scale (cognitive factor).	Over 1/3 of the sample screened positive for minor/major depression (EPDS >10). 16.1% screened positive for major depression (EPDS >13). 10.1% endorsed SI. Sleep onset insomnia and short sleep associated with SI. Obesity, depression and high negative perinatal nocturnal rumination associated with endorsing SI in multivariate analysis. Those experiencing insomnia higher odds of endorsing depression and SI.
Knettel *et al*. (2020) Tanzania	Exploring patterns and predictors of suicidal ideation among pregnant and postpartum women living with HIV in Kilimanjaro, Tanzania.	To examine patterns and predictors of suicidal ideation among women living with HIV in antenatal care in Kilimanjaro, Tanzania.	Longitudinal cohort study. No statistical package reported.	200	Edinburgh Postnatal Depression Scale (EPDS). Patient Health Questionnaire-9 (PHQ-9), Brief Symptom Inventory, Illness Cognition Questionnaire, Beliefs about Medicines Questionnaire, Related Shame Inventory, Perceived Availability of Support Scale (PAS), Norbeck Social Support Questionnaire, Patient-Provider Relationship Scale (PPRS).	Non partnered relationships, unknown HV status of infants father, anxiety, HIV shame/stigma, negative attitudes toward ART, low social support identified in univariate analyses. Interpersonal violence and negative attitudes towards pregnancy significant in multivariate analyses. Anxiety, HIV stigma/shame in significant in final adjusted model.
Kim *et al*. (2015) USA	Suicide risk among perinatal women who report thoughts of self- harm on depression screens.	To estimate the incidence and clinical significance of suicidal ideation revealed during perinatal depression screening and estimate.	Retrospective cohort study. SAS v 9.2.	22,118	Edinburgh Postnatal Depression Scale (EPDS).	Younger age, non-Caucasian race, speaking a non-English language, having public insurance, having a pre-existing mental health diagnosis were highlighted in univariate analyses for antepartum SI. Relationship status, language and relationship status by language interaction, race, sum of EPDS for antepartum SI after multivariate analyses. Unpartnered relationship status, non-Caucasian race, non-English language, public insurance, pre-existing mental health diagnosis and lower neonatal birth weight for postpartum SI. Relationship status, language, relationship status by language interaction, race, EPDS9, caesarean delivery and severe perineal laceration in final analyses for postpartum SI.
Martini *et al*. (2019) Germany	Predictors and outcomes of suicidal ideation during the peri-partum period.	To investigate sociodemographic, gynaecological and clinical predictors of peri-partum suicidality.	Post-hoc analysis. Chi-square. Mann-Whitney u-tests. STATA 14.	306	Composite International Diagnostic Interview for women, Edinburgh Postnatal Depression Scale (EPDS), Brief Symptom Inventory, Social Support Questionnaire.	Peri-partum suicidality likely among women with low household income and a history of childhood abuse/rape, a history of suicide attempt and a history of recurrent depressive and anxiety disorders. Also more likely in women with major depression in the peri-partum period. Increasing maternal age, social support and living with partner decreased odds. Multivariate analysis revealed history of suicide attempt remained associated with peri-partum suicidality and living with partner and social support significantly reduced odds.
Mauri *et al*. (2011) Italy	Suicidality in the perinatal period: comparison of two self-reported instruments. Results from PND-ReScU	To assess suicidality in a non-clinical sample during pregnancy and in the postpartum period. To assess suicidality in women with major and minor depressive episodes during pregnancy and postpartum period.	No study design reported. SPSS 15.	500	Edinburgh Postnatal Depression Scale (EPDS), Mood Spectrum Self-Report (MOODS-SR).	During pregnancy point prevalence of suicidality was highest at 6 months. Postpartum, point prevalence was highest at 3 months.
Molla *et al*. (20220 Ethiopia	Prevalence and associated factors of suicidal behaviour among pregnant mothers in southern Ethiopia: a cross-sectional study.	To assess the prevalence and associated factors of suicidal behaviour among pregnant mothers from the community in the Gedeo zone, southern Ethiopia.	Cross-sectional study. EPI-Data v 3.1 SPSS v 20.	525	Oslo-3, Edinburgh Postnatal Depression Scale (EPDS), Alcohol, Smoking, Substance Involvement Screening Test.	Being unmarried, gestational age of greater than 27 weeks, chronic medical illness, depression and IPV were statistically significant in multivariate analyses.
Muzik *et al*. (2016) USA	Longitudinal suicidal ideation across 18-months postpartum in mothers with childhood maltreatment histories.	To extend understanding of postpartum SI in the context of childhood maltreatment.	Longitudinal study. SPSS v 22. Anova.	116	Postpartum Depression Screening Scale, Childhood Trauma Questionnaire, Shame Attributions Questionnaire, Family, Adaptation, Partnership, Growth, Affection and Resolve Scale, Connor-Davidson Resiliency Scale.	Emotional abuse was the most prevalent of childhood maltreatment. Most participants experienced multiple types of abuse. Endorsement of SI was highest at 4 months postpartum. 28% endorsed SI once. 37% endorsed SI multiple times. 34% never endorsed SI. 11% endorsed SI at all assessments. Severity did not significantly vary between assessments indicating severity was consistent. SI amplified in the presence of childhood maltreatment histories. Parenting a toddler may confer continued stressors for mothers beyond the early infancy period.
Palagini *et al*. (2019) Italy	Stress-related sleep reactivity is associated with insomnia, psychopathology and suicidality in pregnant women: preliminary results.	Pregnant women with high sleep reactivity would report higher symptoms of insomnia, anxiety and depression. Highly sleep reactive pregnant women would be more likely to endorse suicidality compared to women with low sleep reactivity. Explore associations among sleep reactivity, insomnia and depression and anxiety during mid-pregnancy.	No study design reported. NCSS (2008) Shapiro Wilk Test. Mann-Whitney u-test.	62	Ford Insomnia Response to Stress Test., Insomnia Severity Scale, Edinburgh Postnatal Depression Scale (EPDS), Zung Self-rating Anxiety Scale.	High sleep reactivity = more likely to screen positive for depression. Highly reactive sleepers = more likely to endorse anxiety & SI when compared to low reactivity sleepers. No statistically relevant findings for socioeconomic data or gestational week. Univariate logistic regression models identify greater levels of sleep reactivity, depressive symptoms and insomnia are associated with increased odds of endorsing suicidality.
Paris *et al*. (2009) USA	Postpartum depression, suicidality and mother-infant interactions.	Anticipated many women with PPD experienced significant suicidality. Anticipated those with greater distress would feel more suicidal. Higher scores for suicidality would be associated with negative self-appraisal, mothering, the infant and mother-infant relations. Mothers with higher suicidality scores would be less reactive to the needs of their infants.	Mixed methods. SPSS v16.0.	32	Post-Partum Depression Screening Scale, Brief Symptom Inventory, Maternal Self-report Inventory (short form), Parenting Stress Index (short form), Coding Interactive Behavioural Manual.	Single identifiable risk factor for PPD is a mood disorder. 62.5% of mothers had history of depression. 53% recently commenced medication for depression. No differences observed between high/low suicidality on history of depression or medication use. Higher rates of suicidal thoughts related to sleeping and eating problems, feeling more anxious, more labile, more confused, greater loss of self and greater guilt about the experience. Significantly higher severity of overall struggles attributable to PPD in high suicidality group. High suicidality group had greater scores on psychoticism, OCD subscales and indicated greater distortions in thinking and cognitions. More interpersonally sensitive and more somatic symptoms
Pope *et al*. (2013) Canada	A prospective study of self-harm and suicidal ideation during the postpartum period in women with mood disorders.	To explore the prevalence of suicidal ideation during late pregnancy and the postpartum period in women with a history of major depressive disorder or bipolar II disorder.	Prospective study. R v 2.13.1. Student t-test. Fishers exact test.	147	Edinburgh Postnatal Depression Scale (EPDS), Hamilton Depression Rating Scale (HDRS), Young Mania Rating Scale, Structured clinical interview.	Positive relationship between total HDRS score and SI indicating higher levels of depression, more likely to endorse SI. Hypomanic symptoms during pregnancy = more likely to endorse SI. SI more likely in those with bipolar, not statistically significant.
Rodriguez *et al*. (2017) South Africa	Prevalence and psychosocial correlates of suicidal ideation among pregnant women living with HIV in Mpumalanga Province, South Africa.	To estimate the prevalence of and identify risk factors for SI among pregnant WLHIV to inform the development /improvement of clinical care intervention programs and providing pro-grammatic recommendations to access, manage, and treat SI.	No study design reported. SPSS v 21. Mann-Whitney tests. Students t-test. Chi-square test. Fishers exact tests.	673	Edinburgh Postnatal Depression Scale (EPDS), Conflict Tactics Scale Male Involvement Index.	Physical IPV, stigma and depression highlighted in multivariate analyses.
Rodriguez *et al*. (2018) South Africa	Correlates of suicidal ideation during pregnancy and postpartum among women living with HIV in Rural South Africa.	To identify risk factors for suicidal ideation in WLHIV during pregnancy, and to assess their evolution, including continued suicidal ideation into the postnatal period, as well as the emergence and cessation of postnatal suicidal ideation.	Data was drawn from longitudinal RCT. Mann-Whitney test. Mplus v. 7.4.	681	Edinburgh postnatal Depression Scale (EPDS)– 10.	Psychological and physical IPV, depression, stigma and nonadherence identified in bivariate analyses as significant for SI. Antenatal SI continued to be reported at 12 months (n = 26). New-onset SI reported by (n = 51). Increased income, greater stigma associated with the emergence of SI at 12 months for those who had not reported SI at baseline. Cessation of SI (n = 77). Younger age, greater stigma, disclosure of HIV status to partner related to cessation of SI. Physical IPV and depression related to sustained SI (baseline and 12 months).
Shi *et al*. (2018) China	Maternal depression and suicide at immediate prenatal and early postpartum periods and psychosocial risk factors.	To observe maternal depression and suicidal ideation at immediate prenatal (1 week before childbirth) and early postpartum stages (3 and 7 days postpartum), as well as the causal relationship between them. To explore the possible psychosocial risk or protective factors of maternal depression or suicidal ideation at perinatal stage, give guidance for clinical practice in health care professionals at community or hospital.	Longitudinal survey. Paired t-test. No statistical package reported.	271	Edinburgh Postnatal Depression Scale (EPDS), Rosenberg Self-esteem test (REST), Life events scale for pregnant women (LESPW), Pregnancy Pressure Scale (PPS), Social Support Scale (SSS).	Marital dissatisfaction was reported by more mothers experiencing SI. Mothers with SI had relatively higher self-esteem. More mothers experiencing SI had previous miscarriages. Mothers with SI were older, had higher prenatal SI and higher rates of depression.
Shigemi *et al*. (2020) Japan	Suicide attempts among pregnant and postpartum women in Japan: A nationwide retrospective cohort study.	To describe the demographic and clinical characteristics associated with suicide attempts among pregnant and postpartum women in Japan who have psychoneurological disorders.	Retrospective cohort design. Stata v 15.0.	3,286	Not applicable.	Depression was significantly higher among women with suicide attempts in the postpartum. Smoking, psychosis, panic disorder, epilepsy were higher among pregnant women with suicide attempts. Pregnant women with suicide attempts were likely to be younger whereas postpartum women were more likely to be older.
Shigemi *et al*. (2021) Japan	Suicide attempts during pregnancy and perinatal outcomes.	To investigate the patient characteristics and critical perinatal outcomes among pregnant women. To examine the association between methods of suicide attempts and critical perinatal outcomes.	Retrospective cohort study. STATA v 15.0. Students t-test. Mann-Whitney U test.	319	Not applicable.	Depression, schizophrenia and personality disorders were associated with suicide attempts. Depression was more common in the first and second trimesters whilst schizophrenia was more common in late pregnancy.
Sit *et al*. (2011) USA	Seasonal effects on depression risk and suicidal symptoms in postpartum women.	To explore the seasonal variation in risk for depression and SI in postpartum women.	Prospective cohort study. Fishers Exact test. Chi-square. No statistical package reported.	9339	Edinburgh Postnatal Depression Scale (EPDS).	Frequency of suicidal symptoms rise when women had an EPDS score of >15. Frequency of positive EPDS scores was highest in December and lowest in July. No significant year-to-year effect on positive EPDS SCORES.
Spuznar *et al*. (2020) USA	Suicidal ideation in pregnant and postpartum women veterans: an initial clinical needs assessment.	To prospectively characterise hopelessness, depression and suicidality in this population. To explore the relationship between post-traumatic stress symptoms and suicidality.	No study design reported. Correlational analyses. Students t-tests. No software package reported.	23	Columbia Suicide Severity Rating Scale, Becks Hopelessness Scale, Edinburgh Postnatal Depression Scale (EPDS), PTSD Checklist for DS-M V, Life-events checklist from DSM V.	53.6% of participants reported lifetime SI. Since learning they were pregnant, 7.1% experienced a passive level of SI (no intent/plan), 3.6% active SI (some intent/no plan). No suicidal behaviour was reported during pregnancy. No active SI reported during postpartum assessments. Intensity of lifetime SI significantly correlated with PPD but not with symptoms during pregnancy. Posttraumatic stress syndrome not significantly correlated with SI severity at either time.
Supraja *et al*. (2016) India	Suicidality in early pregnancy among antepartum mothers in urban India.	To determine the prevalence of suicidality (suicidal ideation, planning and attempts) during early pregnancy and identifying associated and predictive factors among women attending a public health antenatal clinic.	Cross-sectional study. ANOVA. SPSS v 22.0.	462	Edinburgh Postnatal Depression Scale (EPDS), Suicide Behaviours Questionnaire Revised (SBQ-R).	Bivariate analyses: Women reporting SI were younger. Women from middle socioeconomic status were more likely than women from low socioeconomic status to report SI. Education, parity and employment were not associated with SI. Domestic violence (physical, psychological or sexual), history of suicidality (SI, plans and attempts) were significantly associated with SI. Poor social support was more likely in women with SI. Total EPDS score was higher for women with SI during pregnancy. Multivariate analyses: Age, SES, depressive symptoms, perceived social support, experience of IPV/domestic violence and past history of suicidality were predictors of SI.
Tabb *et al*. (2013) USA	Views and experiences of suicidal ideation during pregnancy and the postpartum: findings from interviews with maternal care clinic patients.	To identify salient themes among a non-treatment sample of perinatal women regarding beliefs about suicidal ideation. Describe experiences with care-seeking for perinatal suicidal ideation and the role that clinical comorbidities play in these beliefs and experiences.	Longitudinal study. Thematic network analysis.	14	Patient Health Questionnaire-9 (short form).	Findings relevant to somatic symptoms were highlighted by women. Poor coping was identified such as self-harm, acting out, isolating behaviours and substance and tobacco use.
Tabb *et al*. (2019) USA	Prevalence of antenatal suicidal ideation among racially and ethnically diverse WIC enrolled women receiving care in a Midwestern public health clinic.	To determine the prevalence of SI and risk factors associated with SI during pregnancy among a sample of low-income pregnant women enrolled in a WIC programme.	Cross-sectional study. Chi-square. Student t-test. STATA 14.2.	736	Edinburgh Postnatal Depression Scale (EPDS).	N = 34 women endorsed SI. A greater proportion of this group were smokers. 20.6% experiencing SI reported undergoing a pregnancy termination prior to current pregnancy but not considered statistically significant. Every one-point increase on EPDS score observed a 39% increase in the odds of reporting SI, unadjusted. Smoking status, education, income and age constant for every one-point increase in the EPDS score the odds of reporting SI increased by 43%.
Zewdu *et al*. (2021) Ethiopia	Prevalence of suicidal ideation and associated factors among HIV positive perinatal women on follow-up at Gondar town health institutions, Northwest Ethiopia: a cross-sectional study.	To determine the prevalence of suicidal ideation and associated factors among HIV positive perinatal women in the study setting.	Cross-sectional study. EPI INFO v 7. STATA v 14	422	Edinburgh Postnatal Depression Scale (EPDS), Oslo Social Support (OSS-3)	Perinatal depression, unplanned pregnancy and non-disclosed HIV status were factors associated with SI in multivariable analyses. Unplanned pregnancy odds of SI were 2.75 times higher than those with planned pregnancy. Undisclosed HIV status–odds 3.73 times higher than those who disclosed their status. Perinatal depression odds of SI 4.40 times higher than those with no perinatal depression.
Zhang *et al*. (2020) China	Suicide ideation among pregnant women: The role of different experiences of childhood abuse.	To examine the association between childhood abuse, including emotional abuse, physical abuse, and sexual abuse, and suicide ideation among pregnant women in the general population in China.	No study design reported. SPSS v 21.0.	1825	Childhood Trauma Questionnaire (CTQ), Patient Health Questionnaire-9 (PHQ-9).	Pregnant women with any experience of childhood abuse history have high risk of SI. Pregnant women with only emotional abuse, only physical abuse or any two abuses were at higher risk of SI. Women with childhood abuse and no antenatal depression had a 2.57 fold higher risk of SI. Women with childhood abuse and antenatal depression had a 6.72fold higher risk of SI. Women with childhood abuse and depression had a 17.78fold higher risk of SI.
Zhong *et al*. (2016) Peru	Childhood abuse and suicidal ideation in a cohort of pregnant Peruvian women.	To assess the extent women’s history of physical and/or sexual abuse in childhood is associated with antepartum suicidal ideation.	Cross-sectional study. ANOVA. SAS version 9.4. R v 3.1.0.	2964	Childhood Physical and Sexual Abuse Questionnaire, Patient Health Questionnaire-9 (PHQ-9).	Those who experienced childhood abuse had an increased risk of SI irrespective of depression status.

### Biological risk-factors for suicidal ideation in the perinatal period

There were specific biological risk factors evident across several areas (see Table 3) identified as being positively associated with perinatal suicidal ideation; sleep, maternal age, lifestyle factors, genetics and pregnancy-related complications. Sleep deprivation was identified as being associated with higher rates of suicidal ideation [34]. Utilising the Postpartum Depression Screening Scale (PDSS) [75] those in a high suicidality group scored 18.47 on sleeping and eating subscales compared to 12.33 in the low suicidality group [34]. Those with poor subjective sleep quality were 2.85 times more likely to experience suicidal ideation when compared to women who had good sleep quality [47]. Similarly, insomnia and rumination were identified as being associated with suicidal ideation [45]. Using the Insomnia Severity Index (ISI) [76] participants with insomnia experienced suicidal ideation at a rate of 15.5% (n = 13/84), this was compared to those who did not experience insomnia at a rate of 7.7% (n = 14/183). Furthermore, participants experiencing high sleep reactivity (defined as difficulty falling or staying asleep even after the removal of stressors) were more likely to report higher levels of insomnia and were therefore more likely to screen positive for depression and to experience suicidal ideation [57].

**Table 3:** Biological risk factors.

*Biological risk-factors for suicidal ideation in the perinatal period*	
Sleep deprivation / insomnia (n = 4)	Paris *et al*. (2009); Palagini *et al*. (2019); Kalmbach *et al*. (2020); Anbesaw *et al*. (2021)
Maternal age (n = 4)	Howard *et al*. (2011); Gressier *et al*. (2016); Supraja *et al*. (2016); Martini *et al*. (2019)
Smoking (n = 2)	Tabb *et al*. (2013); Gressier *et al*. (2016)
Alcohol (n = 1)	Tabb *et al*. (2019)
Pregnancy conditions (n = 3)	Girardi *et al*. (2011); Kim *et al*. (2015); Kalmbach *et al*. (2020)
Birth complications (n = 2)	Tabb *et al*. (2013); Bodnar-Deren *et al*. (2016)
Infant illness (n = 1)	Belete & Misgan (2019)
Traumatic experiences/pregnancy loss (n = 2)	Gressier *et al*. (2016); Tabb *et al*. (2019)
Chronic medical conditions (n = 3)	Rodriguez *et al*. *(*2018); Zewdu *et al*. (2021); Molla *et al*. (2022);
Accessed termination services (n = 2)	Tabb *et al*. (2019); Anbesaw *et al*. (2021)
Dysregulated immunity (n = 1)	Achtyes *et al*. (2020)

Participants with chronic medical conditions were 4.47 times more likely to experience suicidal behaviours compared to those without chronic medical conditions [50]. Biological changes such as inflammation and low levels of serotonin were also found to be associated with suicidal ideation, possibly indicating dysregulated immunity in women with postpartum depression [44]. An association between younger maternal age and suicidal ideation was also identified as a biological risk factor [63, 64, 71]. Increasing maternal age was associated with decreased odds of suicidal ideation [52, 66].

Lifestyle factors such as smoking and alcohol use were identified as being positively associated with perinatal suicidal ideation. Women reported using tobacco and substances as a means of escaping difficult situations [37] however, the study does not report whether these are prescribed or illicit substances. Pregnant women (n = 34) experiencing suicidal ideation were more likely to be smokers (n = 8) [43]. This was also highlighted in a retrospective study of 1439 women, where tobacco use during pregnancy was associated with an increased risk of experiencing suicidal ideation in the perinatal period [64]. Of this sample, 154 women attempted suicide in the perinatal period; 49 in pregnancy and 105 postpartum [64]. This study compared three groups: women with suicide attempts in pregnancy, women with suicide attempts in the postpartum and women without suicide attempts. Smoking was measured at 57.14% and 36.08% compared to 36.51% of women who had no suicide attempts [64]. Alcohol use was measured in the same study at a rate of 18.37% and 8.51% respectively compared to 7.83% for women who had no suicide attempts [64].

Physical pregnancy-related risk factors also played a role in increasing the risk of suicidal ideation in the perinatal period. Women who experienced severe perineal laceration, caesarean delivery [36], severe hyperemesis gravidarum (3.2%), pregnancy-induced hypertension (9.7%), gestational diabetes mellitus (6.5%) [56] and obesity [45] were reported to be at increased risk of suicidal ideation. In addition, pregnancy and birth complications, birth trauma and infant illness were reported as being associated with suicidal ideation [37, 39, 46]. Molla *et al*. [50] reported women with a gestational age of greater than 27 weeks were 4.92 times more likely to experience suicidal behaviours, compared to those with a gestational age of less than 14 weeks. This cross-sectional study examined suicidal behaviour in pregnant women to integrate mental healthcare with maternal healthcare [50]. These findings were mirrored by du Toit *et al*. [54] who reported women presenting before 13 weeks gestation were less likely to experience suicidal ideation (IR = 1.2) when compared to those presenting later than 27 weeks’ gestation (IR 6.8); these findings were identified as independent risk factors after multiple regression analyses. Furthermore, traumatic experiences relating to pregnancy loss were also identified as an independent risk factor [64]. Women experiencing suicidal ideation also reported to have accessed termination services for previous pregnancies [43, 47], however this was not considered statistically significant [43].

### Psychological risk factors associated with suicidal ideation in the perinatal period

Psychological risk factors were evident across several areas (see Table 4) and identified as being positively associated with perinatal suicidal ideation to include miscarriage, mental health problems and trauma. The psychological sequelae following miscarriage was identified as attributing to increased risk of suicide attempts in the perinatal period [64]. Subsequent pregnancies, especially those occurring shortly after a miscarriage created stress and uncertainty for the woman by reactivating trauma [64]. In addition, women who felt less prepared for motherhood were more likely to experience negative implications of maternal mental wellbeing [34]. Of note was the reporting of the fact that childbirth may be an anti-climactic event for some women which compounds low mood [37] and may induce negative attitudes towards the pregnancy [73]. Furthermore, unplanned pregnancy was seen to increase the risk of women endorsing suicidal ideation by 2 times [48] to 2.75 times [49]. In multivariate analyses, unplanned pregnancy remained a significant factor [46, 72]. Interestingly, du Toit *et al*. [54] also reported unplanned pregnancies as a risk factor but only for women who were unemployed.

**Table 4:** Psychological risk factors.

*Psychological risk factors for suicidal ideation in the perinatal period*	
Miscarriage (n = 3)	Paris *et al*. (2009); Tabb *et al*. (2013); Gressier *et al*. (2016)
Preparedness for motherhood (n = 2)	Paris *et al*. (2009); Tabb *et al*. (2013)
Negative attitudes towards pregnancy (n = 1)	Knettel *et al*. *(*2020)
Unplanned pregnancy (n = 5)	Belete & Misgan (2019); du Toit *et al*. (2020); Fellmeth *et al*. (2021); Zewdu *et al*. (2021), Belete *et al*. (2021);
Mental health problems (n = 20)	Asad *et al*. (2010); Ammerman *et al*. (2019); Doherty *et al*. (2019); du Toit *et al*. (2019); Martini *et al*. (2019); Pope *et al*. (2013); Alhusen *et al*. (2015); Girardi *et al*. (2011); Tabb *et al*. (2013); Muzik *et al*. (2016); Bodnar-Deren *et al*. (2016); Rodriguez *et al*. (2017); Shi *et al*. (2018); Enatescu *et al*. (2020); Shigemi *et al*. (2020); Anbesaw *et al*. (2021); Belete *et al*. (2021) Shigemi *et al*. (2021); Zewdu *et al*. (2021); Molla *et al*. (2022)
History of suicide attempt (n = 4)	Asad *et al*. (2010); Supraja *et al*. (2016); Ammerman *et al*. (2019); du Toit *et al*. (2019)
Personality (n = 2)	Enatescu *et al*. (2020); Gelabert *et al*. (2020)
Traumatic experiences (n = 8)	Muzik *et al*. (2015); Zhong *et al*. (2016); Shi *et al*. (2018); Ammerman *et al*. (2019); Belete & Misgan (2019); Martini *et al*. (2019); Gelabert *et al*. (2020); Spuznar *et al*. (2020)

Mental health problems such as adjustment disorder, bipolar affective disorder, depression and anxiety were identified as contributing to suicidal ideation in the perinatal period, particularly in the context of existing mental health problems. Women with a diagnosis of adjustment disorder experienced higher levels of suicidal intent, but this was not considered statistically significant [74]. Moreover, women with a history of suicide attempts [42, 54, 70, 71] anxiety disorders [66, 68, 70], stress [47], depressive disorders [42, 51, 54, 58, 60, 61, 68, 70], bipolar affective disorder [65] or post-traumatic stress disorder [42] were at increased risk of suicidal ideation during the perinatal period. Women with a diagnosis of depression were between 2.32 times [50], 4 times [47] and 4.40 times [49] more likely to experience suicidal ideation. Similarly, Belete *et al*. [48] found women to be 3 times more likely to experience suicidal ideation compared to women who had not experienced common antenatal mental health disorders. Those with hypomanic symptoms during the postpartum period were more likely to experience suicidal ideation however this finding was not statistically significant [65]. Indeed, lifetime suicidal ideation meant women were 4.6 times more likely to experience suicidal ideation during their current pregnancy [48].

Women with suicidal ideation showed higher scores for certain personality dimensions such as neuroticism [67, 68] and psychoticism [67] which indicates greater distortions in thinking. Borderline personality disorder and traits were highlighted as being positively associated with perinatal suicidality [54]. Of note, diagnoses such as generalised anxiety disorders and schizophrenia spectrum disorders were associated with a lower risk of suicidal ideation [54], conversely, schizophrenia was more common in women with suicide attempts in later pregnancy (28–26 weeks gestation) [61].

The use of the EPDS [77] identified depressive symptoms and suicidal ideation in 22.89% (n = 38) of women during their current pregnancy and of that number, 97.3% achieved scores of >12 [38]. Furthermore, dysphoric/dysregulated temperaments were identified in 52% of women that had an EPDS score of >13 compared to women with low temperament traits at 8% [56]. Of relevance was the reporting of the fact that for every one-point increase on an EPDS score, there was a 39% increase in the odds of reporting suicidal ideation [37]. Timeframes associated with suicidal symptom severity ranged across studies; during pregnancy the sixth month was the most prevalent [40, 61] and after pregnancy the point prevalence was highest at three months [40], four months [40] and approximately six months [39]. EPDS scores were higher for women experiencing suicidal ideation antenatally when compared to those who were not experiencing suicidal ideation [36, 71]. Of note, women who reported suicidal ideation on the EPDS antenatally, 7% (n = 26) continued to report suicidal ideation at 12 months [52] however, 19% (n = 77) reported to have no suicidal ideation postnatally [52]. The Hamilton Depression Rating Scale (HDRS) [78] identified 59.7% (n = 52) of participants experiencing suicidal ideation had moderate or severe depressive symptoms where the risk of suicide was 1.17 times greater with every one-point increase in HDRS scores [53]. This longitudinal study examined the association between perinatal depressive symptoms and suicide risk among low-income women in South Africa [53].

The psychological impact of traumatic experiences during pregnancy was attributed to perinatal suicidal ideation [58, 67]. In addition, 7.2% (n = 2) of women veterans who had experienced a lifetime traumatic event reported passive levels of suicidal ideation (no intent/plan), 3.6% (n = 1) reported active suicidal ideation (some intent/no plan) during pregnancy however, no active suicidal ideation was reported during postpartum assessments [41] which indicates giving birth may be a protective factor. Women who experienced childhood trauma [42] and maltreatment, particularly maltreatment at the hands of their parents [40] were also at increased risk of perinatal suicidal ideation. Of note were women who experienced childhood abuse and/or rape who were more likely to experience perinatal suicidality (Mean = 6, SD 40.0) compared to those who did not (Mean = 241, SD 88.9) [66]. Women who experienced childhood abuse were 2.57 times more likely to experience suicidal ideation and this increased to 17.78 times more likely if the woman also had a diagnosis of depression [59]. There was also a positive association between the number of childhood abuse incidences; women who experienced greater than 6 episodes of abuse were 5.3 times more likely to experience suicidal ideation [62]. Similarly maternal abuse (verbal, sexual and physical) was identified in 52.8% of women who experienced suicidal ideation [46]. Women living with HIV and who perceived stigma were also likely to experience suicidal ideation [51, 73]. Indeed, participants living with HIV who had not disclosed their status were 3.73 times more likely to experience suicidal ideation compared to those who had disclosed their status [49]. The non-disclosure of HIV status was attributed to perceived stigma and a loss of social support. This finding was mirrored by Rodriguez *et al*. [52] who identified women that had disclosed their HIV status to their partners experienced a cessation of suicidal ideation.

### Social risk factors associated with suicidal ideation in the perinatal period

Social risk factors were evident across several areas (see Table 5) and identified as being positively associated with perinatal suicidal ideation and include employment status, household income, education, refugee status and the presence of intimate partner violence (IPV). Women who were unemployed were more likely to experience suicidal ideation than those engaged in active employment [54, 63, 68], however this risk factor was not statistically significant in the postpartum period [68]. Supraja *et al*. [71] report employment status was not identified as a risk factor in their study. Furthermore, suicidal ideation was more likely among women with low household incomes [66] particularly those with an annual income of less than $10,000 [38]. Women with less than 12 years formal education were also more likely to screen positive for suicidal ideation [38]. Lower education levels were statistically significant (*p* = .041) for women presenting with suicidal ideation in the postnatal period [68]. Interestingly, education was not seen as a significant risk factor by Asad *et al*. [70] or Supraja *et al*. [71]. This cross-sectional study examined suicidality in antepartum mothers in urban India [71]. Refugee women were also more likely to experience suicidal ideation than migrant women indicating that refugee status may be associated with a greater sense of hopelessness [72].

**Table 5:** Social risk factors.

*Social risk factors for suicidal ideation in the perinatal period*	
Unemployment (n = 3)	Howard *et al*. (2011); du Toit *et al*. (2019); Enatescu *et al*. (2020)
Low income(n = 2)	Alhusen *et al*. (2015); Martini *et al*. (2019)
Education (n = 2)	Alhusen *et al*. (2015); Enatescu *et al*. (2020)
Poor relationships (n = 3)	Tabb *et al*. (2013); Alhusen *et al*. (2015); Muzik *et al*. (2015)
Refugee status (n = 1)	Fellmeth *et al*. (2021)
Absence of close relationship or support network (n = 4)	Alhusen *et al*. (2015); Muzik *et al*. (2015); Kim *et al*. (2015); Anbesaw *et al*. (2021)
Intimate Partner Violence (IPV) (n = 7)	Asad *et al*. (2010); Supraja *et al*. (2016); Rodriguez *et al*. (2017); Islam *et al*. (2019*);* Knellel *et al*. (2020); Anbesaw *et al*. (2021); Molla *et al*. (2022)

Stressful and dysfunctional relationships can increase the risk of suicidal ideation in the perinatal period [37, 58]. IPV was reported as a significant indicator of risk [51, 69, 73]. Indeed, 20% (n = 32) of women experienced abuse during pregnancy with an odds ratio of between 3.41–25.75 when depressive symptoms, socio-demographic factors and the presence of IPV were analysed [38]. Molla *et al*. [50] also reported women who experienced IPV were 7.60 times more likely to experience suicidal behaviour whilst Anbesaw *et al*. [47] identified IPV as increasingly the likelihood of experiencing suicidal ideation however, the risk was lower at 2.45 times. This cross-sectional study examined antenatal suicidal ideation, and this may be a factor in the reduced risk. Furthermore, the rate of domestic violence was 42.9% (n = 15) for women who experienced suicidal ideation [71]. The frequency at which women experienced physical and sexual abuse was also positively associated with suicidal ideation; 43% of women exposed to physical/sexual abuse more than once per week experienced suicidal ideation compared to 13% of women who experienced physical/sexual abuse less than once per month [70].

Conversely, living without a partner was seen to increase the risk of perinatal suicidality [36, 38] a finding mirrored by Anbesaw *et al*. [47] where women who were single, widowed or divorced were 2.8 times more likely to experience suicidal ideation when compared with women who were married. Belete and Misgan [46] report that 62.3% of women endorsing suicidal ideation had sexually unfaithful husbands. Poor interpersonal relationships and diminished support networks in the perinatal period were believed to compound maternal stress levels and therefore increase the risk of suicidal ideation [40, 42, 58] whilst higher perceived social support was associated with decreased risk of suicidal ideation [71].

## Discussion

**Fig 2:**
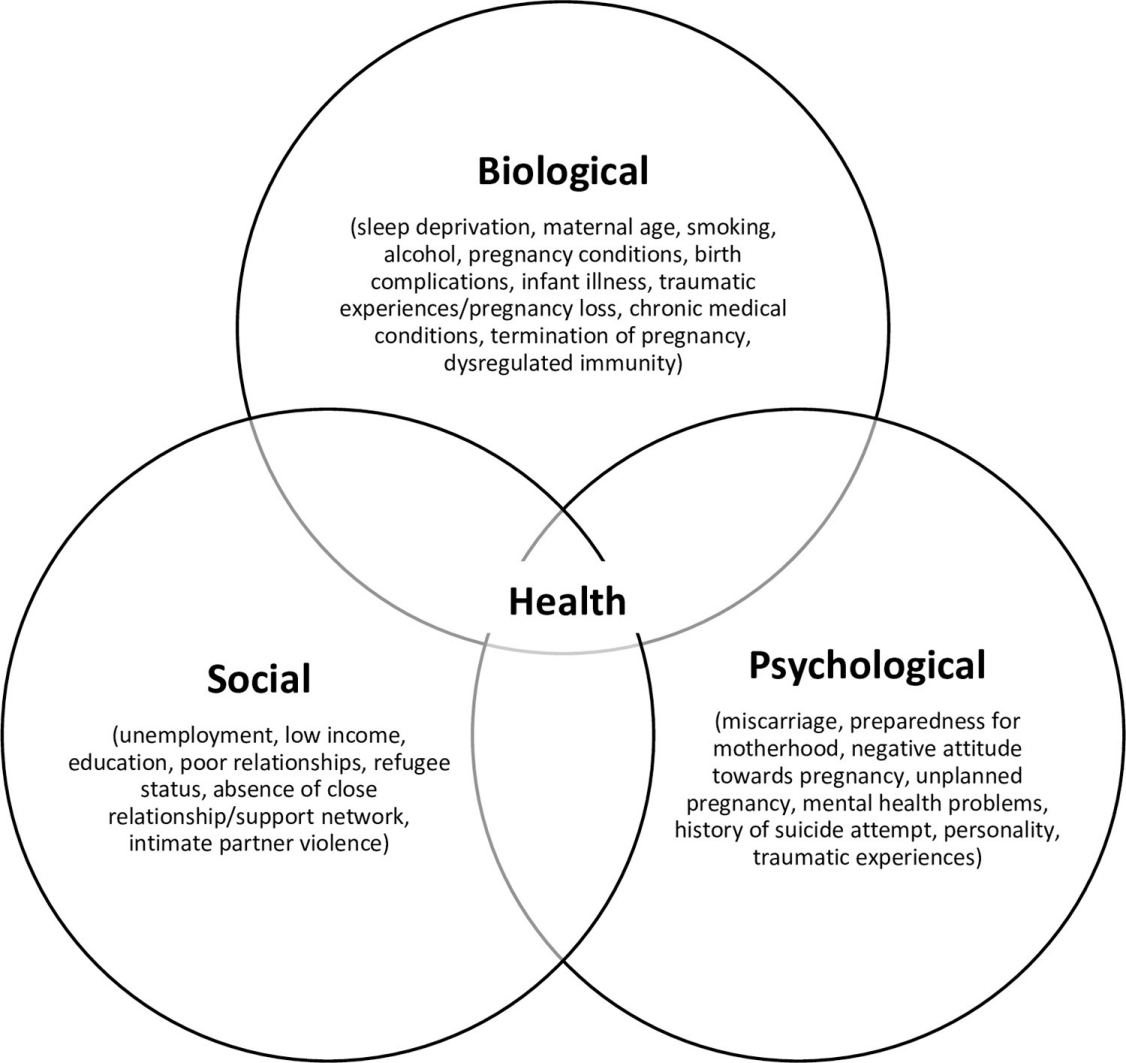
Biopsychosocial framework (Engel 1977).

This review identifies the existing literature and the range of biological, psychological and social risk factors associated with suicidal ideation in the perinatal period. Forty-one articles were identified, and findings mapped onto the biopsychosocial framework (see Fig 2) [33]. Utilising the biopsychosocial framework [33] laid bare the interplay between each of the identified risk factors, many of which were applicable to multiple categories. For healthcare practitioners, the results of this review are of particular importance as it demonstrates the need for biopsychosocial assessment and care of women in the perinatal period and the need for sensitive enquiry about suicidal ideation with women presenting with the identified risk factors. Furthermore, the results of this review emphasise that problems are multifaceted and therefore interventions must also provide person-centred benefit to ensure better clinical outcomes.

Within the biological risk category, this review highlights a lack of quality sleep is associated with increased risk of suicidal ideation and this may be the result of depressive symptoms where sleep is disturbed [34] and insomnia or rumination [45, 57]. Sleep deprivation can lead to emotional lability, confusion, a distorted sense of self, it can induce abnormal neural responses that may alter reward processing capabilities [65, 79] and in a general sense, a link exists between insomnia and the development of mental health problems [80]. There is also an association between sleep deprivation and obsessive-compulsive disorder in the perinatal period [81]. Existing literature in the context of sleep deprivation in new mothers highlights the negative impacts on emotional and physical wellbeing [82, 83]. Gelayea *et al*. [84] add to these findings by highlighting the correlation between poor sleep and perinatal suicidal ideation. With the review findings aligning with what is currently known about poor quality sleep and perinatal suicidal ideation, this raises the question as to whether sleep is seen as a clinical priority by healthcare practitioners in the context of maternal mental wellbeing? Or is there a tendency for healthcare professionals to overlook the importance of sleep, as lack of sleep may be attributed to being a ‘normal’ experience in the perinatal period? Sleep can impact a person across the biopsychosocial spectrum and is a modifiable risk factor for perinatal suicidal ideation. It is important poor-quality sleep is seen as a clinical priority and that adequate enquiry is made with women in the perinatal period about their sleep quality. Therefore, it is recommended that healthcare professionals make specific enquiry about sleep and not solely rely on the results of question seven of the EPDS [77]. Furthermore, it is recommended that healthcare professionals provide advice to women to ensure good sleep hygiene and sleep promotion practices are utilised to optimise their sleep [85]. For women experiencing poor quality sleep because of mental health problems, treatment will include education, monitoring, CBT or medication, dependent on symptom severity [85]. The perinatal period results in increased demands both physically and mentally on mothers, therefore by ensuring good quality sleep is achieved, this may mitigate any risk associated with consistently inadequate sleep.

Unsurprisingly, within this review under the psychological risk factor category mental health problems were highlighted as being associated with perinatal suicidal ideation, particularly for women with pre-existing mental health problems or those who develop mental ill-health during pregnancy. Mood and mental health problems and their sequelae can impact not only psychological but biological and social facets of the lives of women. The existing literature in the context of mental ill-health during pregnancy suggests women in the perinatal period are more vulnerable to mental health problems and suicidal ideation [22] and it is also accepted those with a diagnosis of bipolar affective disorder or schizophrenia are more likely to complete suicide during pregnancy [86]. Furthermore, the psychological impact of historical abuse and trauma were also identified as being positively associated with perinatal suicidal ideation [40–42, 59, 62, 66, 67] and may manifest as symptoms consistent with post-traumatic stress such as avoidance, negative alterations in mood and thinking, emotional dysregulation and negative self-perception [87]. There is once again, demonstrated alignment with what is known about maternal mental ill-health and suicidal ideation therefore, this raises two important questions; is adequate enquiry into maternal mental health made during the perinatal period? and what guidance do healthcare professionals, who have limited mental health expertise/training have to help them determine if such enquiries are needed? Firstly, there is some evidence to suggest that enquiries into maternal mental health are less than robust [1] indicating a need for greater clarity surrounding maternal mental health enquiry. Secondly, international best practice guidelines conclude enquiry about depressive symptoms should be made at minimum on booking visit, postnatally at four to six weeks and again at three to four months and that screening tools should be used at least once in the perinatal period [88, 89]. However, there is limited evidence to suggest screening for perinatal depression improves outcomes [89].

The most used screening tool in the included studies was the EPDS [77]. Despite highlighting the presence of depressive symptoms, the EPDS is not a diagnostic tool and does not indicate severity or duration of depressive symptoms, puerperal psychosis or bipolar affective disorder nor should it be used on a pass/fail basis [77, 90]. The results of the EPDS should be considered as part of a comprehensive clinical interview which seeks to gather information about the individual across the biopsychosocial and life spectrum such as individual history, family history and social and employment history [91] and should be carried out by competent healthcare professionals so that results are interpreted correctly and a person-centred plan of care can be established if required. The use of screening tools are seen as a way to ‘open the conversation’ surrounding mental health. Therefore, in most instances enquiry into maternal mental health may be initiated using the Whooley questions [92], a practice supported by best-practice guidelines [93]. There are also promising results on the use of digital applications such as the MGH Perinatal Depression Scale (MGHPDS) which collects self-report data on relevant risk factors such as mood, anxiety, sleep and stress and may help to increase the identification of perinatal mental ill-health and subsequent access to mental health services [94]. This, in addition to inter-disciplinary working with colleagues in mental health would help to ensure adequate assessment, interpretation and interventions are planned to support women experiencing mood and mental health problems during the perinatal period and to mitigate any risk associated with mental ill-health during the perinatal period. The findings of this review highlight that trauma can take many forms and may present as adverse childhood experiences (ACE’s), psychological trauma, physical trauma and sexual trauma. In instances where trauma history is reported, it is essential women receive specialist, high quality interventions from trained professionals to manage these symptoms whilst also being signposted to peer support networks [87].

It is interesting that this review also identifies miscarriage as associated with perinatal suicidal ideation [64]. In the context of perinatal loss, women may experience more profound reactions when compared to men and the bereavement process may be complex [95, 96]. Perinatal grief is often referred to as disenfranchised as the loss cannot be publicly mourned [97]. This may lead to the development of complicated grief characterised by the suppression or voiding of emotions over a protracted period [12]. Grief reactions are known to be exacerbated by existing vulnerabilities and susceptibilities therefore it is important perinatal grief reactions are not minimised [98]. However, it is important to note that some women will have dealt with the loss and are subsequently not vulnerable. It is recommended healthcare practitioners obtain a comprehensive obstetric history to include previous perinatal losses which will alert them to existing vulnerabilities and will enable them to plan care accordingly which may include additional monitoring or referral to specialist services.

Within the social risk factors category of this review, low socioeconomic status was positively associated with perinatal suicidal ideation, particularly in the context of employment [54, 63, 68], income [66] and education [38, 68]. Having a low socioeconomic status is positively linked to poorer health outcomes [99]. In a general sense, deprivation, poverty and inequality are linked to mental health difficulties with financial stress also known to be a risk to mental wellbeing [100, 101]. As outlined by the WHO [101] health is determined on many levels and is not limited to mental and physical wellbeing but is also determined by adequate income, access to health services, social inclusion, education and social capital. This raises an important question; how can healthcare professionals best intervene when socioeconomic problems are identified during assessment? With the exception of referral to social supports, the healthcare professional is powerless to help owing to a lack of resources and time. However, there are ways in which indirect support can be provided by facilitating events such as parent coffee mornings or parent and baby groups, liaising and building good working relationships with local charities, organisations and baby banks that help those from low socioeconomic backgrounds. Again, this inter-professional working may help to ensure women have access to a range of services and will demonstrate to women that help is available to those who seek it. To be aware of what additional supports are on offer, the healthcare professional must familiarise themselves with what is available in the local community, but this can take time and may put added pressure on healthcare providers who are already working under severe pressure. Additional support is particularly important for those women with low social capital, who are in strained relationships or who have little support from family and friends–this type of support is necessary for human interaction, wellbeing and socialisation and also helps to ensure adequate rest and sleep and therefore impacts across the biopsychosocial spectrum. However, whilst it is accepted that low socioeconomic status, mental ill-health and poor relationships may increase the risk of perinatal mental health problems, healthcare practitioners must maintain an open and enquiring approach to assessment so as not to make assumptions or stigmatise women with these risk factors. Therefore, it is recommended healthcare practitioners provide a non-judgemental space that provides adequate time and opportunity to open conversation about women’s needs, experiences and expectations of the perinatal period. Here, the healthcare practitioner can respond by actively listening and giving credence to the women’s narratives. Often, this process in itself is cathartic and may be enough to inspire hope in a woman who may feel hopeless. If during this time the woman was provided with non-judgemental support and information, it may empower the woman to seek further support if her mental health deteriorated, giving her a sense of autonomy and control congruent with the principles of recovery [102].

### Implications for research and practice

The findings from this review indicate there is an urgent need for further qualitative research to be carried out with women experiencing perinatal suicidal ideation to get a deeper understanding of their experiences. There is also a need for further research to be conducted with women experiencing perinatal suicidal ideation in conjunction with their partners to capture their experiences and to help identify why partner support is seen to be a protective factor from perinatal suicidal ideation. Future systematic reviews should focus on sleep practices and the impact of sleep on perinatal suicidal ideation. In addition, systematic reviews on the role of the social determinants of health may help to identify how these factors contribute to suicidality.

As the features of mental health crises can be exacerbated by myriad different triggers unique to the individual [12] and when one considers those who attempt or complete suicide in the perinatal period may not be known to mental health services [103], it is recommended those working in primary and maternity care become familiar with safety planning and mental health crisis care pathways to disseminate information to women whether they are deemed at risk or not. It is recommended women with existing mental health needs be facilitated with pre-conception planning or advice in the context of their mental health to discuss any concerns relating to medication and to ensure women are informed of symptoms relating to relapse and/or crisis which will help facilitate a prompt response. If the woman is already involved with mental health services, this should be addressed by their mental health team to maintain continuity of care within an already established therapeutic relationship. Healthcare practitioners must acknowledge that pregnancy, birth and miscarriage or perinatal loss may be traumatic, and women should be afforded psychological support in relation to these experiences should they wish to avail of them. Therefore, it is recommended healthcare practitioners respond with empathy and compassion, without feeling the need to ‘fix’ as by doing so may minimise the experience for the woman. Finally, it is recommended that greater inter-professional working between those in mental health services and primary care/maternity services is facilitated to ensure women have access to specialist, trained healthcare professionals without the need for additional referral.

### Strengths and limitations

One significant strength of this review is that it is the only scoping review the authors are aware of in the context of perinatal suicidal ideation. Furthermore, it reinforces the need for holistic assessment of women in the perinatal period as suicidal ideation is not limited to psychological factors. Second, the broad range of countries represented in the dataset demonstrates findings are similar in this context in both high and low-income countries. However, this strength can also be a limitation as there may be other compounding variables relating to perinatal suicidal ideation such as cultural considerations relevant to the findings. Furthermore, only studies published in English were considered and this may have led to an unavoidable language bias. Inclusion criteria with a greater date range may have yielded more relevant publications. As this is a scoping review, we did not assess the methodological quality of the included studies, and this should be taken into consideration when interpreting the findings. Finally, while an information specialist was not consulted in the generation of search strategy the author team has extensive searching and review experience.

## Conclusion

This review of the biopsychosocial risk factors associated with perinatal suicidal ideation has identified numerous and varied considerations spanning the entire biopsychosocial spectrum with some having overlapping impacts on more than one area. The findings of this review identified the following risk factors are associated with perinatal suicidal ideation: mental ill-health (existing and new onset), poor quality sleep, intimate partner/domestic violence, history of childhood maltreatment/abuse, alcohol/tobacco use, trauma (physical / emotional / sexual), low socioeconomic status and miscarriage/perinatal loss.

The perinatal period is a time of great change and transition for the woman and her family that leaves her vulnerable to the development of mental health problems. It is therefore essential that healthcare practitioners working with women in the perinatal period are cognisant that women have needs of their own, existing outside the parameters of pregnancy that are often exacerbated by pregnancy itself. It is for this reason sensitive enquiry into maternal mental health needs to be made with every woman, irrespective of how she presents to ensure no woman slips ‘through the cracks’ [104]. Those working with women in the perinatal period must be sensitive to the impact of poor-quality sleep and ensure to provide advice on improving sleep quality and duration. Healthcare professionals must also ensure to open conversations with women in the context of mental health in such a way as to elicit candid reports of their experience that does not stigmatise or judge but facilitates effective therapeutic relationships.

## Supporting information

S1 Checklist PRISMA-SR checklist. (PDF)
